# Supplementary material for: Predicting high-fitness viral protein variants with Bayesian active learning and biophysics
Source: Proc Natl Acad Sci U S A. 2025 Jun 9;122(24):e2503742122. doi: 10.1073/pnas.2503742122 (PMC12184641; doi:10.1073/pnas.2503742122)
Supplement: Supplementary file 1 — Appendix 01 (PDF) [file pnas.2503742122.sapp.pdf]

PNAS

Supporting Information for

Few Shots Viral Variant Detection via Bayesian Active Learning and Biophysics

Marian Huot, Dianzhuo Wang, Jiacheng Liu, and Eugene I. Shakhnovich

Dianzhuo Wang and Eugene I. Shakhnovich.
E-mail: johnwang@g.harvard.edu, shakhnovich@chemistry.harvard.edu

This PDF file includes:

Supporting text
Figs. S1 to S5

Supporting Information Text

Active learning on DMS.

We assess the capacity of our pipeline combined with active learning to identify high fitness single variants in the DMS dataset (Figure S1). This task requires to model the impact of approximately $193 * 19$ mutations, with initial knowledge of only one mutation per site. Interestingly, the UCB acquisition perform best in early rounds, while greedy acquisition gets better as the predictor gets more accurate on the longer term. This is explained by the high accuracy of our model to identify top variants after a few round of acquisitions, with AUC above 0.80.

Greedy acquisition on CM dataset.

We show the performance of the greedy acquisition strategy declines as we tighten the definition of dangerous variants by lowering the threshold p (Figure S2). This effect is particularly pronounced during intermediary rounds. Unlike UCB acquisition, the acquired mutants under the greedy strategy are not preferentially enriched near the 99% fitness quantile. As a result, when p decreases, the number of top-fitness variants identified drops sharply, highlighting the limitations of greedy acquisition in stringent discovery settings.

Influence of uncertainty weight in UCB acquisition. Increasing the uncertainty weight β in UCB acquisition enhances embedding variance and higher diversity in the training set and potentially improving predictive performance (higher AUC) (Figure S3). However, in the long run, as the predictor becomes more accurate, it is preferable to reduce the emphasis on uncertainty, allowing the model to rely more on its predictions for optimal decision-making. Consequently, we selected $\beta = 0.2$ as a reasonable trade-off between exploitation and exploration of the mutational landscape. However, it is important to note that the impact of β on overall performance remains relatively limited, and one can choose any reasonable β *a priori* for the purpose of pandemic prevention.

β is then scaled by $\frac{\text{std(fitnesses)}}{\text{std}(\sqrt{\text{vars}})}$ to ensure that the uncertainty term $\sqrt{\text{vars}}$ has a comparable range to the fitness values.

Semantic change in CM dataset.

We observe that variants with more mutations relative to the wild-type exhibit greater semantic change (Figure S4). Since UCB prioritizes variants with higher predictive uncertainty—initially correlated with high semantic change—it tends to be biased early on toward acquiring variants with a larger number of mutations.

Identification of mutation-prone sites.

We assessed site mutability by analyzing amino acid diversity at each position within the RBD (Figure S5). In the main text, sites were labeled as "highly mutable" if they exhibited at least 9 distinct amino acid variants out of the possible 20, observed in mutants with a count of 10 or more. As demonstrated in Figure S3, the performance of VIRAL in identifying highly mutated sites remains robust across different thresholds defining mutation frequency (from >4 to >11 mutations), confirming the consistency of our approach regardless of the specific cutoff chosen for analysis.

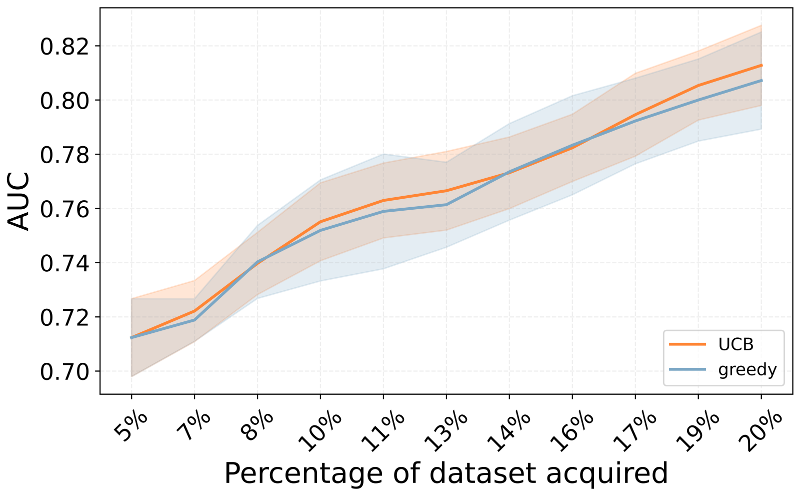

(a)

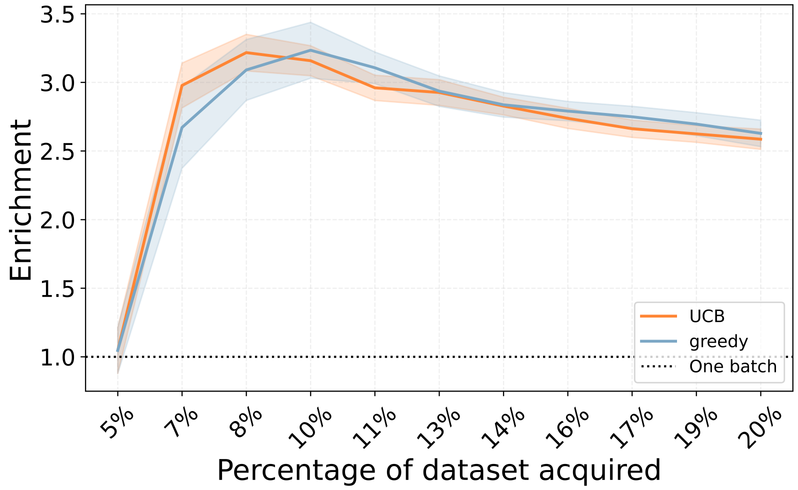

(b)

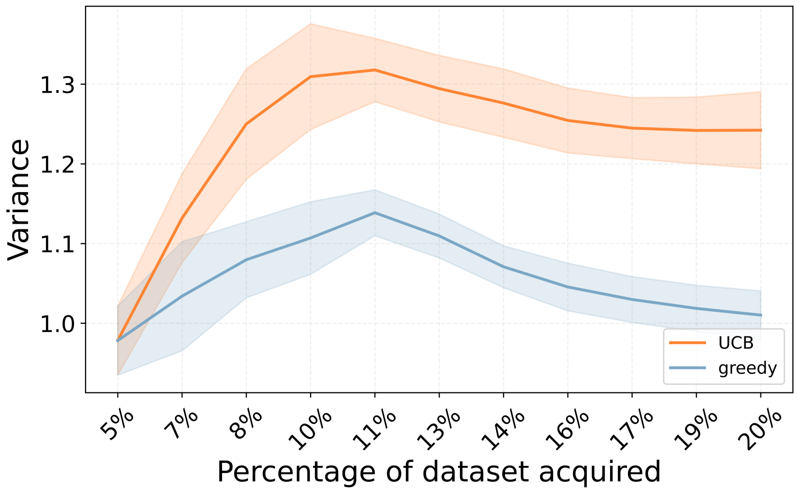

(c)

Fig. S1. Active learning on DMS. (a) Area Under the Curve (AUC), representing the model's ability to identify top fitness variants, is shown for different strategies (UCB and greedy). An AUC above 0.5 indicates effective identification of high-fitness variants. Each round corresponds to acquiring a new batch of variants, improving the predictor. Shaded regions represent standard deviations across runs. (b) Enrichment in top variants across acquisition runs for each strategy. Values above 1 represent improvement other a random acquisition. (c) Comparison of embedding variance of points acquired across rounds between UCB (orange) and greedy (blue) strategies

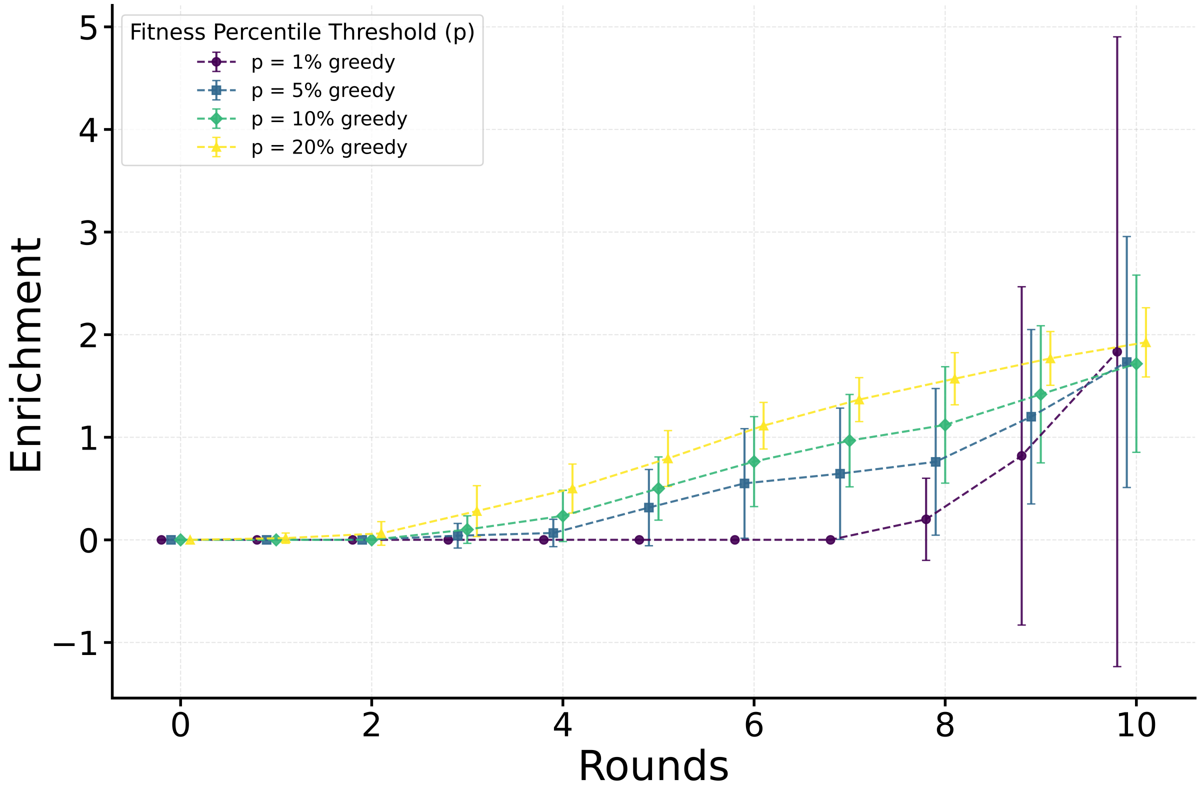

(a)

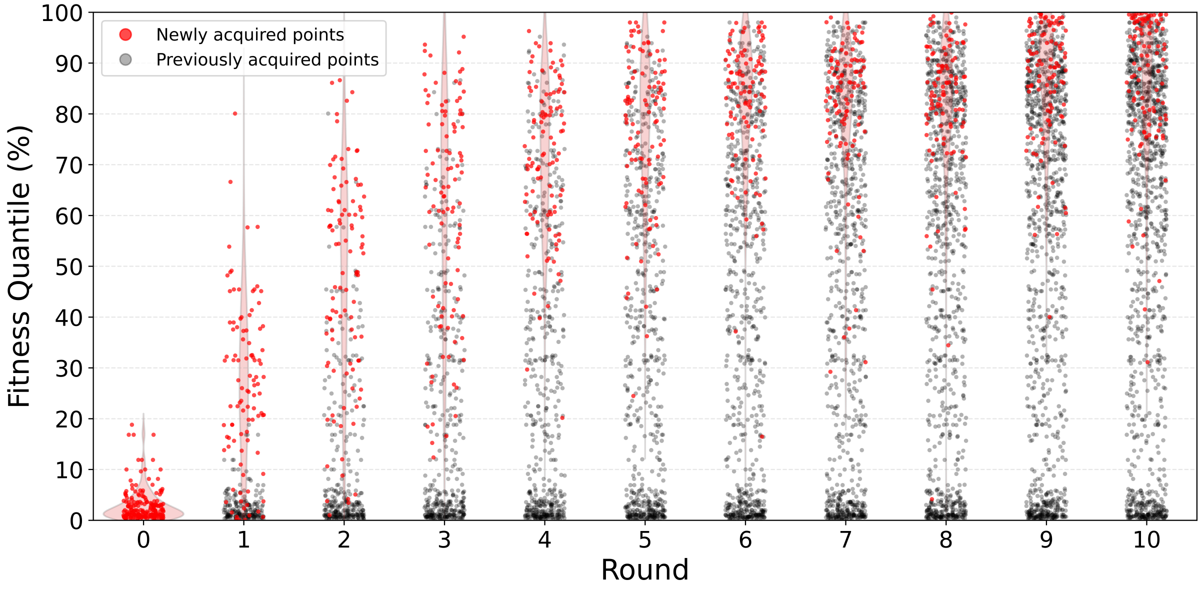

(b)

Fig. S2. Active learning on CM dataset with greedy acquisition. (a) Enrichment across acquisitions runs for different fitness thresholds p defining top variants. (b) Fitness of acquired variants at every round. New acquired variants are shown as red dots.

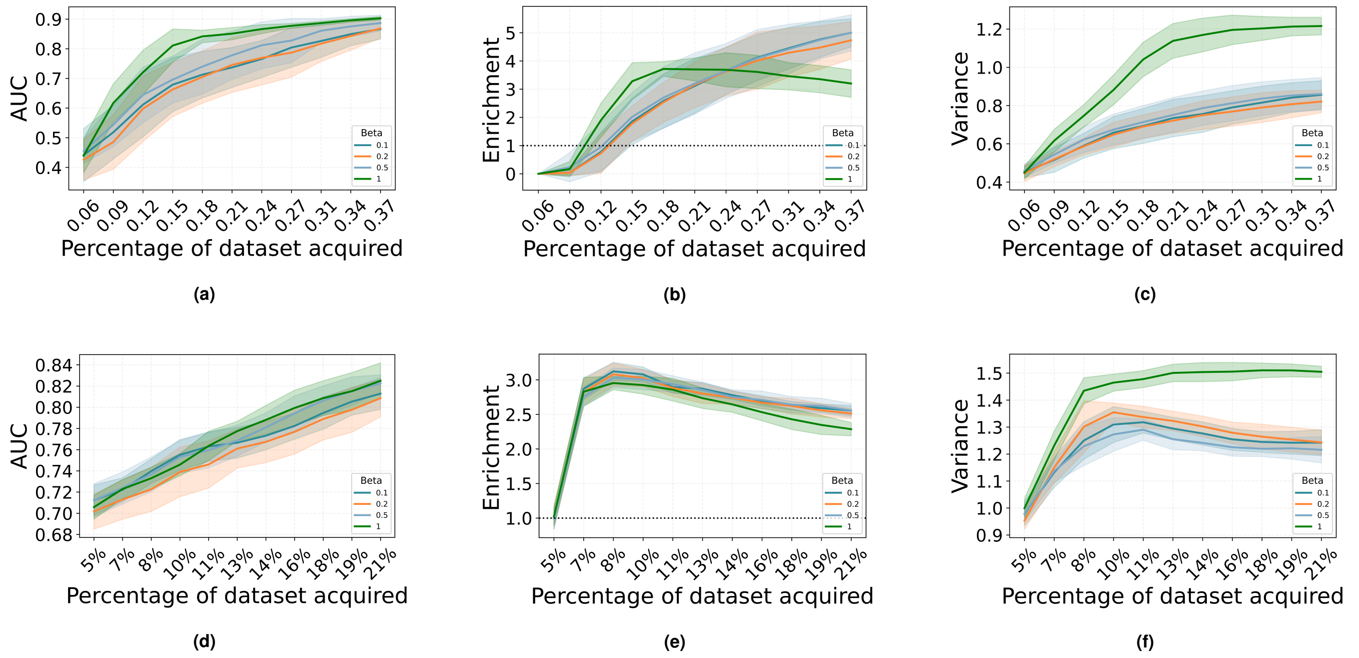

Fig. S3. Influence of uncertainty weight β in UCB acquisition. (a-c) AUC, enrichment, and embedding variance on the CM dataset. (d-f) Similar analysis on the DMS dataset. Different values of β control the balance between exploration and exploitation, affecting model performance in identifying high-fitness variants.

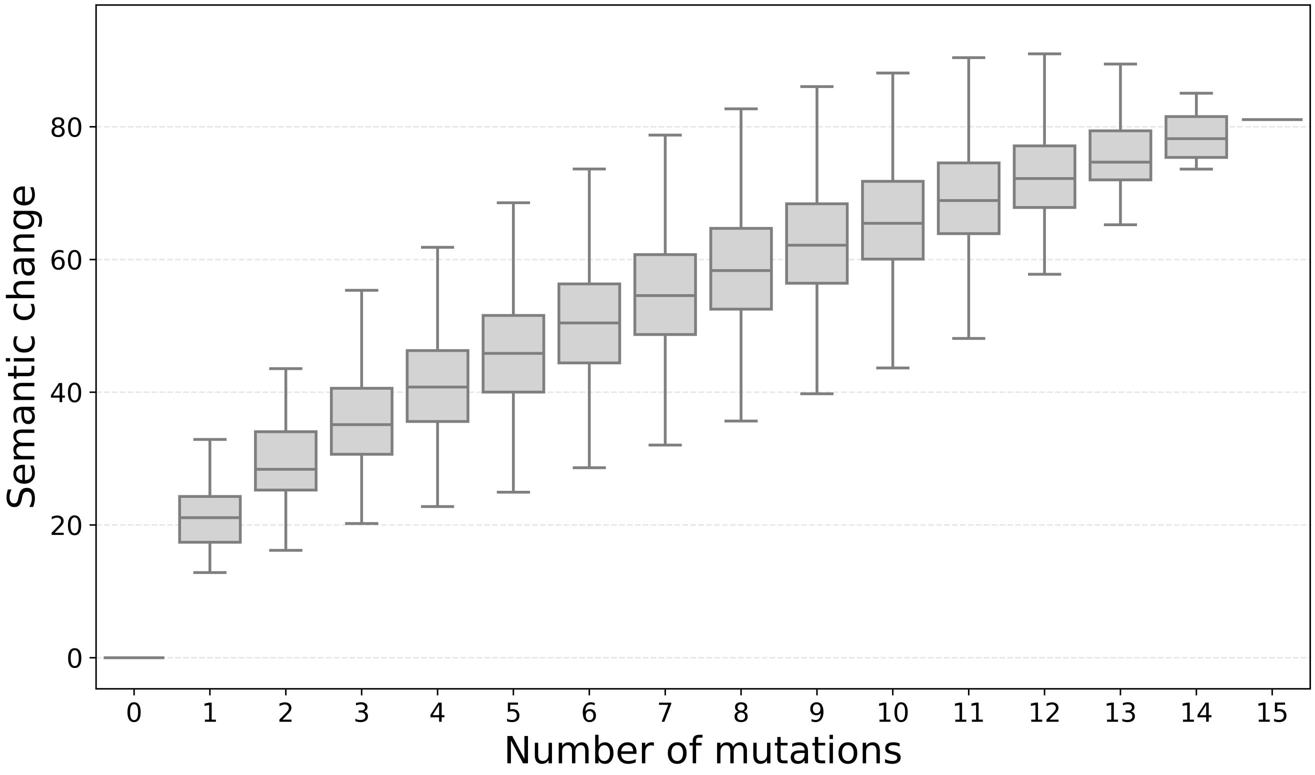

Fig. S4. Semantic change vs. number of mutations in the CM dataset.

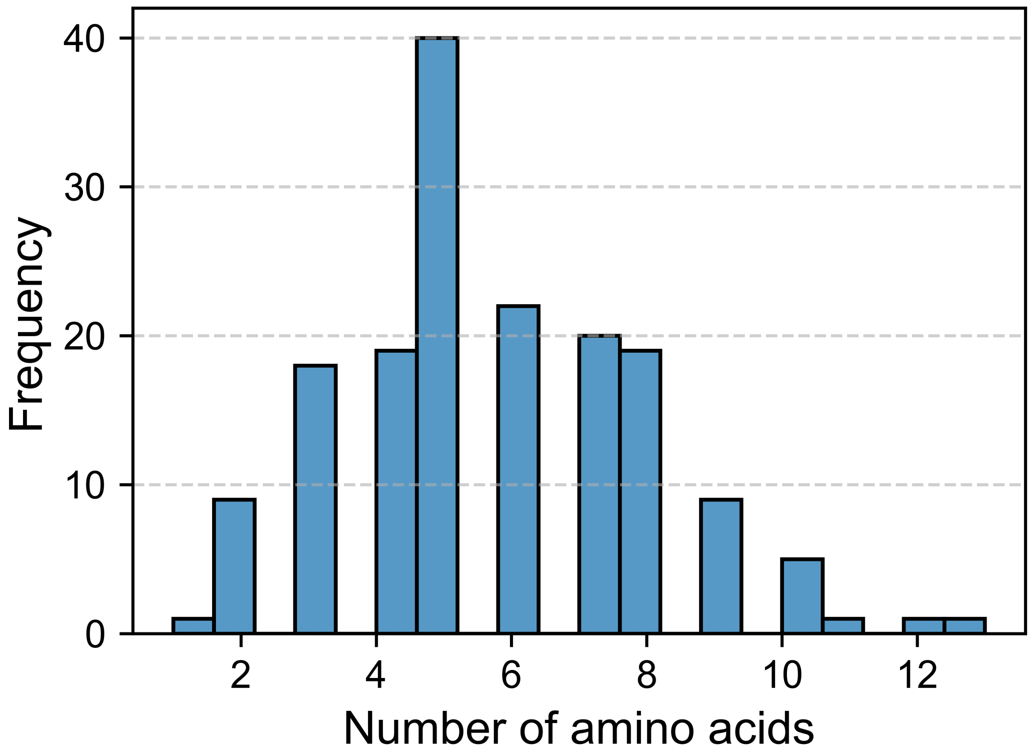

(a)

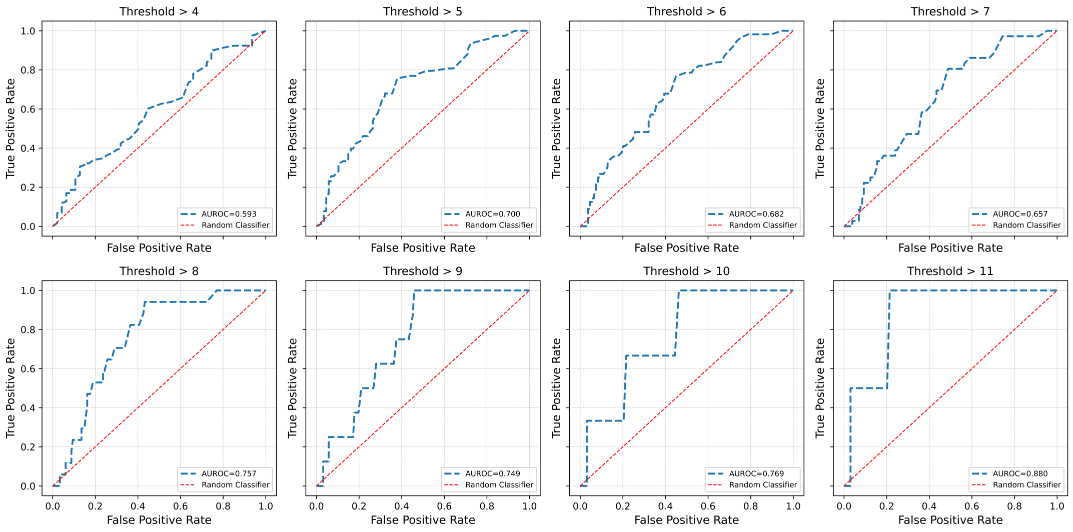

(b)

Fig. S5. Active learning performance for identifying mutation-prone sites across different thresholds. (a) Histogram showing the distribution of SARS-CoV-2 RBD sites by the number of distinct amino acid substitutions observed during the pandemic. The data reveals most sites experienced between 4-8 different amino acid changes, with a peak at 6 amino acid variants. (b) ROC curves demonstrating VIRAL's ability to identify highly mutable sites in the GISAID database across various thresholds (from >4 to >11 mutations). The averaged acquisition scores from 10 independent runs show consistently strong predictive performance, with AUC values ranging from 0.593 to 0.890. Higher thresholds (>9, >10, >11) yield the best performance (AUC>0.75), indicating VIRAL is particularly effective at identifying the most highly mutable positions in the viral genome.
